# Next-generation Bioinformatics: connecting bases to genes, networks and disease

**DOI:** 10.1093/bib/bbt090

**Published:** 2014-03-14

**Authors:** Paul Horton

In this issue, we present a collection of papers inspired by ISCB-Asia/SCCG 2012, an international conference on bioinformatics co-organized by the International Society of Computational Biology and BGI, known for their Sequencing Center near the conference site in Shenzhen, China.

The issue begins at the whiteboard fastidiously counting bits, gradually moves into the more abstract world of inferring various kinds of gene networks and ends near the clinic reviewing methods to detect diabetes biomarkers and cancer driver mutations. In the first paper, Shrestha *et al.* give the reader a look ‘under-the-hood’ at how to efficiently construct suffix arrays, a data structure forming the basis for most methods used in the initial processing of high-throughput sequencing data. In the second paper, Gromiha & Ou review databases, online resources and sequence analysis techniques to characterize membrane proteins primarily from their amino acid sequence. In the third paper, Vreven *et al.* compare methods to predict protein–protein complexes from sequence.

The next three papers focus on the inference of various kinds of networks. B. Chen *et al.* review methods for inferring protein–protein interaction networks from a collection of (possibly error-prone) protein–protein interaction pairs. Maetschke *et al.* show a little teaching can go a long way in their comparison of supervised versus unsupervised inference methods for gene regulatory networks. Kim *et al.* provide a comprehensive review of methods for inferring dynamic networks from time course data. Both B. Chen *et al.* and Kim *et al.* discuss the decomposition of interactions in spatial (subcellular localization) and temporal dimensions.

The last two papers focus on medical applications. Li *et al.* review the dynamical network biomarkers method to identify genes marking pre-disease to disease transition from time course expression data, applying the method on animal models of type 2 diabetes as a test case. Finally, Zhang *et al.* bring us back to the genome by reviewing methods to distinguish driver mutations from passenger mutations in cancer.

Despite the broad range of topics covered, several issues are encountered in multiple papers. Among the common issues are low concordance between different prediction methods, high variance in performance across samples and the difficulties in constructing realistic and informative performance benchmarks. Another common thread is the need to integrate highly diverse sources of information to produce biologically relevant results.

One clear conclusion is that we have a long road ahead of us before our discipline fully catches up to the potential insights, which high-throughput sequencing promises. Even simply constructing an efficient data structure to hold sequence data is nontrivial, predicting a single interaction pair challenging, and automatically inferring genome-scale gene networks is currently infeasible. However, this issue also gives us a glimpse of what is waiting for us at the end of that road—improved health for mankind.

